# Supplementary material for: Implementation of the My Abilities First Tool: A qualitative study on the perceptions of professionals, caregivers, children, and adolescents with disabilities
Source: PLoS One. 2024 May 20;19(5):e0301718. doi: 10.1371/journal.pone.0301718 (PMC11104674; doi:10.1371/journal.pone.0301718)
Supplement: Attachment — Submitted filename: Response to Reviewers.docx [file pone.0301718.s002.docx]

**RESPONSE TO EDITOR PONE-D-23-41828**

Renato S. Melo, PhD

Academic Editor

PLOS ONE

On behalf of all the authors, I would like to express my gratitude for the opportunity to revise and resubmit the manuscript [PONE-D-23-41828], entitled 'Implementation of the My Abilities First Tool: a qualitative study on the perceptions of professionals, caregivers, children, and adolescents with disabilities', for publication in PLOS ONE. We greatly appreciate and value the time and attention you and the reviewers dedicated to providing feedback on our work. We are particularly grateful for the insightful comments that have led to significant improvements. We have meticulously incorporated the suggestions provided by you and the reviewers.

At this time, we are confident that our responses sufficiently address the comments provided, and we are prepared to finalize the revised version of the manuscript, taking into consideration any additional suggestions that the reviewers may offer. We have attached all responses pertaining to the suggestions for the resubmission.

Below we have provided our specific point-by-point responses to the editor's and reviewers' comments and new suggestions, highlighted in blue. All proposed modifications and corrections have been accepted by all authors and incorporated into the manuscript to elevate its standard and quality.

We hereby certify that all authors have thoroughly reviewed the manuscript, confirm its originality, and affirm that it is neither under consideration nor published, either wholly or partially, in another journal or similar publication. All authors unanimously consent to the submission of this article to PLOS ONE.

Please do not hesitate to contact me as the corresponding author.

Roselene Ferreira de Alencar

Department of Physical Therapy.

Federal University of Rio Grande do Norte,

Natal, Rio Grande do Norte, Brazil

E-mail: roselene.alencar@ufrn.br

**RESPONSE TO REVIEWER PONE-D-23-41828**

**Comments to the Author**

1. Is the manuscript technically sound, and do the data support the conclusions?

The manuscript must describe a technically sound piece of scientific research with data that supports the conclusions. Experiments must have been conducted rigorously, with appropriate controls, replication, and sample sizes. The conclusions must be drawn appropriately based on the data presented.

Reviewer #1: Yes

Reviewer #2: Yes

Reviewer #3: Yes

**Response from Alencar et al.**

- Thank you for the valuable feedback. We are considering the suggestions carefully while reviewing our manuscript.
- The study was conducted with ethical rigor and, considering it is a qualitative research, the sample was estimated to be a representation of the population, intentionally selected to provide relevant information. As for the conclusions reached, they were appropriately based on the presented data.
- We will always be available to make improvements.

2. Has the statistical analysis been performed appropriately and rigorously?

Reviewer #1: Yes

Reviewer #2: Yes

Reviewer #3: Yes

**Response from Alencar et al.**

- We sincerely appreciate your question. Regarding the statistical analysis, we would like to reiterate that our research was conducted within a qualitative framework, using Reflective Thematic Analysis.

3. Have the authors made all data underlying the findings in their manuscript fully available?

The [PLOS Data policy](http://www.plosone.org/static/policies.action#sharing) requires authors to make all data underlying the findings described in their manuscript fully available without restriction, with rare exception (please refer to the Data Availability Statement in the manuscript PDF file). The data should be provided as part of the manuscript or its supporting information, or deposited to a public repository. For example, in addition to summary statistics, the data points behind means, medians and variance measures should be available. If there are restrictions on publicly sharing data—e.g. participant privacy or use of data from a third party—those must be specified.

Reviewer #1: Yes

Reviewer #2: Yes

Reviewer #3: Yes

**Response from Alencar et al.**

- We sincerely appreciate your question. Yes, we confirm that all data underlying the results described in our manuscript have been made fully available. We are committed to ensuring transparency and access to the data to promote the replicability and integrity of our research.
- The data that have been carefully referenced and presented in the manuscript or stored have undergone all stages of the Reflexive Thematic Analysis process.

4. Is the manuscript presented in an intelligible fashion and written in standard English?

PLOS ONE does not copyedit accepted manuscripts, so the language in submitted articles must be clear, correct, and unambiguous. Any typographical or grammatical errors should be corrected at revision, so please note any specific errors here.

Reviewer #1: Yes

Reviewer #2: Yes

Reviewer #3: Yes

**Response from Alencar et al.**

- We sincerely appreciate your question and comment. We confirm that the manuscript is presented in an understandable manner and written in standard English. We are committed to ensuring clarity and linguistic correctness in our article.
- We will always be available to make improvements.

5. **Review Comments to the Author**

Please use the space provided to explain your answers to the questions above. You may also include additional comments for the author, including concerns about dual publication, research ethics, or publication ethics. (Please upload your review as an attachment if it exceeds 20,000 characters).

**Reviewer #1:** The manuscript presents a comprehensive study on the perceptions of professionals, caregivers, children, and adolescents with disabilities regarding the implementation of the My Abilities First (MAF) tool in Specialized Child Rehabilitation Centers (CERs). Overall, the manuscript makes a valuable contribution to the field of rehabilitation by shedding light on the perceptions of various stakeholders regarding the MAF tool. I have some suggestions and comments designed to improve this manuscript:

Introduction:
-The introduction briefly mentions the MAF tool and its grounding in positive language, valuing the potential of children and adolescents with disabilities. Adding a concise description or definition of the MAF tool would enhance understanding and provide clarity on its intended impact.

**Response from Alencar et al.**

- We thank you for the suggestion for improvement. We agree that a more detailed description of the MAF tool in the introduction could benefit the reader's understanding of the tool's context and clarity about its intended impact.
- We would like to mention that there is also a text about the tool in the Materials and Methods/My Abilities First Tool section **[Ln. 226 - 246, P. 10,12]**.
- In response to this comment, we inserted a concise description of the MAF tool in the Introduction section **[Ln. 90-100, P. 3,4].** We added the reference Liao et al., 2023. Please see below.

*In this regard, the educational tool My Abilities First (MAF), conceived by Verònica Schiariti and disseminated in 2020, aims to achieve these measures of attention to people with disabilities. MAF is grounded in the use of positive language and the process of informing professionals, family members, and the general public about the importance of positive attitudes towards people with disabilities [6]. According to Schiariti (2020a), the use of positive language, associated with the MAF approach, means paying attention to what the person with a disability can do. MAF proposes the adoption of a skills identification card, the My Abilities ID CARD [2,6]. Furthermore, it is a proposal that, by valuing the abilities of children and adolescents with disabilities, can contribute to increased community engagement and effective participation [6,8,9,10].*

Method:
- While the educational interventions are briefly mentioned, providing more details on the content, structure, and objectives of the theoretical and practical workshops would enhance the reader's understanding of their role in the study.

**Response from Alencar et al.**

- We appreciate the observation. We agree that providing more details about the educational interventions is essential for a comprehensive understanding of their role in the study.
- In response to the request, we added a table.
- Please see **Materials and method section/ Educational Interventions [Table 1. Workshop Content, Ln. 255 and 256, P. 10 and 11]**.

-The authors need to further clarify code identification to illustrate the semantic and latent ways of coding to assist in understanding the analytical process more concretely.

**Response from Alencar et al.**

- Thank you for the suggestion. We agree that greater clarity can significantly contribute to a deeper understanding of the analytical process.
- **Data Analysis [Ln. 287 - 294, P. 13 and 14]**.

*The themes were identified through predominantly semantic coding, meaning that the description of the speeches did not require continuous exploration of hidden meanings, due to the clarity of these speeches and the focus on a specific theme, which was the implementation process of the MAF tool. However, there was some latent level coding when it was necessary to employ a more interpretative exercise of the speeches, considering, to justify this need, the participants' premises, context, and social position, such as speeches with the association of regional terms in unfinished sentences.*

-Given the emphasis on reflexive analysis, it would be valuable if the authors include a brief discussion on the researchers' reflexivity – acknowledging and addressing potential biases or preconceptions that may have influenced the research process and outcomes.

**Response from Alencar et al.**

- Thank you for the observation. We recognize the importance of including researchers' reflexivity for the transparency and integrity of the study.
- Please see in the **Discussion section [Ln. 627 - 636, P. 27]**

*The implementation of the MAF tool may face challenges, with resistance to attitude changes being one of the most significant, especially when prioritizing the recognition of the abilities of people with disabilities over a disability-focused approach. This process requires a deeper understanding of the MAF tool and the development of strategies for the effective use of the My Abilities ID CARD, as discussed by Schiariti (2020) and Liao et al. (2023).To overcome these challenges, actions such as continuous education for professionals and health education for healthcare service users, as well as for the general population, play crucial roles. These measures not only support the adoption of the most appropriate strategies for the implementation process but also promote a broader and more inclusive understanding of the abilities of people with disabilities.*

-Providing information on the time allocation for the theoretical and practical workshops would offer insights into the intensity of participant engagement and the depth of knowledge transfer.

**Response from Alencar et al.**

- Thank you for the suggestion. We agree that providing more information about the workshops can enhance understanding of participant engagement and knowledge transfer.
- In response to the request, we added a table.
- Please see **Materials and method section/ Educational Interventions [Table 1. Workshop Content, Ln. 255 and 256, P. 10 and 11]**.

Result:
-Clarifying the total number of participants considered for each percentage would enhance transparency.

**Response from Alencar et al.**

- Thank you. In order to increase transparency, our revised version contains the requested information about the number of participants for each percentage.
- **Results section [Ln. 326 - 332, P. 15]**.

*A total of 11 children/adolescents with their caregivers participated in the study. Diverse sociodemographic and clinical characteristics were recorded, and percentages were calculated based on the numerical values assigned to each question, aiming for better exposure and transparency of the obtained data. The predominant age of the children and adolescents was 7 years (54.5%), and all attended regular education. Further sociodemographic information of this group is available in Table 2.*

-While direct participant quotes are included, providing a more extensive context or explanation for selected quotes would help in interpreting the nuances of participant responses.

**Response from Alencar et al.**

- We appreciate the observation. We recognize that providing a broader context for participant quotes can enrich the understanding of the nuances of their responses.
- Please see in the **Results section [Ln. 540 - 549, P. 24]**.

*The respondents' answers reflect a variety of perceptions about the implementation and usefulness of the MAF tool. While some highlighted potential benefits, such as increased self-esteem, appreciation of individual abilities, and contribution to promoting inclusion, others expressed concerns about possible adverse effects, such as increased discrimination and stigma. There was also a common emphasis on the importance of a person-centered approach, recognizing individual abilities and the importance of active participation of individuals with disabilities in their communities. These diverse perspectives offer insights to guide future research and practices in the field of children and adolescents' rehabilitation.*

-Given the qualitative nature of the study and the use of Reflexive Thematic Analysis, it would be beneficial to include a brief reflection on the researchers' reflexivity and its potential impact on data interpretation.

**Response from Alencar et al.**

- Thank you for the observation. We agree to include the reflexivity of the researchers.
- Please see in the **Discussion section [Ln. 627 - 636, P.27]**

*The implementation of the MAF tool may face challenges, with resistance to attitude changes being one of the most significant, especially when prioritizing the recognition of the abilities of people with disabilities over a disability-focused approach. This process requires a deeper understanding of the MAF tool and the development of strategies for the effective use of the My Abilities ID CARD, as discussed by Schiariti (2020) and Liao et al. (2023).To overcome these challenges, actions such as continuous education for professionals and health education for healthcare service users, as well as for the general population, play crucial roles. These measures not only support the adoption of the most appropriate strategies for the implementation process but also promote a broader and more inclusive understanding of the abilities of people with disabilities.*

Discussion:
-The discussion could benefit from a more detailed exploration of specific challenges encountered during the implementation of the MAF tool, providing insights into practical issues and potential solutions.

**Response from Alencar et al.**

- Thank you for the observation. We have included in the revised version a more detailed exploration of the challenges in the implementation process of the MAF tool.
- Please see in the Discussion section **[Ln. 627 - 636, P.27]**.

*The implementation of the MAF tool may face challenges, with resistance to attitude changes being one of the most significant, especially when prioritizing the recognition of the abilities of people with disabilities over a disability-focused approach. This process requires a deeper understanding of the MAF tool and the development of strategies for the effective use of the My Abilities ID CARD, as discussed by Schiariti (2020) and Liao et al. (2023).To overcome these challenges, actions such as continuous education for professionals and health education for healthcare service users, as well as for the general population, play crucial roles. These measures not only support the adoption of the most appropriate strategies for the implementation process but also promote a broader and more inclusive understanding of the abilities of people with disabilities.*

-While participants emphasize the importance of government actions, more specific policy recommendations or considerations for policymakers could be discussed. This would add depth to the suggestions for promoting broad access and minimizing potential risks.

**Response from Alencar et al.**

- Thank you for the observation and suggestion. We agree that discussing more specific policy recommendations can enrich the study's content.
- Please see in the **Discussion section [Ln. 609 - 615, P.26].**

*The need to instigate governmental actions was expressed in the study. The allocation of government resources can be positive for applying the principles of equity, justice, and respect for human rights. Additionally, it can promote process efficiency, the exchange of knowledge and experiences, as well as ensure an integrated and holistic approach to promoting broad access to health and inclusion with effective participation of people with disabilities, corroborating with proposals by Schiariti and McWilliam (2021) [2] and Schiariti (2020) [6].*

-While the authors mention that the MAF tool is perceived as an innovation, they do not mention what makes it innovative. Needs more explanation.

**Response from Alencar et al.**

- Thank you for your valuable observation. We acknowledge the need to provide a more detailed explanation of what makes the MAF tool innovative.
- **Discussion section [Ln. 556 and 557, P. 24]**.

*The MAF is seen as an innovative tool for emphasizing the individual abilities of people with disabilities, rather than just focusing on their disabilities.*

Reviewer #2: Good Idea.
Some Grammatical mistakes to be corrected and spelling mistake in line 584 and some.

**Response from Alencar et al.**

- Thank you for your feedback. We have carefully reviewed the manuscript to correct any grammatical errors and address the spelling mistake you identified.
- Correction "in **line 584, P. 25**" (original version), in the corrected version **line 604 and 605 P. 26** "In the scope of Theme 2, entitled 'Barriers and Facilitators for the Implementation Process of MAF,' the emphasis was on the importance of *promoting behavioral changes and attitude changes.*

Data Analysis can be depicted in graph or pie chart for each component as it is a qualitative study.

**Response from Alencar et al.**

- Thank you for the suggestion.
- The graphs presented in the manuscript were employed to visualize the collected data. Regarding data analysis, given the qualitative nature of the study focusing on participants' speech, we believe that a descriptive text presentation of its phases would be more appropriate.
- We are available for further guidance.

Limitations and challenges faced during the study can be added.

**Response from Alencar et al.**

- Thank you for the suggestion. We are considering adding the limitations and challenges faced during the study.
- Please in the **Final Considerations [Ln. 663 - 670, P. 28 and 29]**.

*Based on these results and considering the limitations outlined in the study, the lack of greater diversity in the sample in terms of cultural, socioeconomic, and primarily geographical differences, as well as the absence of cross-cultural adaptation of the MAF tool, may have affected obtaining a better perspective on the results. These two aspects may be crucial for promising directions in future research.*

*Furthermore, other important perspectives to be included in future studies include the formulation of strategies for the use of the My Abilities ID CARD and the development of protocols or implementation manuals for the MAF.*

Sample size is small and its a Pilot study and equal no of samples were not taken.

**Response from Alencar et al.**

- Thank you for the suggestion, but our study is a pilot study for the implementation of the MAF tool in Brazil. Nevertheless, we have added this text below to meet the request.
- Please see **Materials and method section/Participants and Eligibility Criteria [Ln. 162-166, P.6]**.

*The mentioned rehabilitation centers accommodated approximately 90 individuals with the potential to participate in the study. All of them were contacted; however, 27 participants met the inclusion criteria and responded to the semi-structured questionnaires and interviews I and II, after signing consent forms and authorization for images and audios.*

Reviewer #3: This study was aimed to the perceptions of professionals, caregivers, children, and 49 adolescents with disabilities regarding the implementation of the My Abilities First 50 (MAF) tool in Specialized Child Rehabilitation Centers (CERs). Authors concluded that The implementation of the MAF tool was 66 perceived as an innovation due to its focus on the abilities of individuals with 67 disabilities. However, there is a need to restructure it to broaden its scope and access 68 to different contexts in order to confront barriers and enhance the inclusion and 69 participation of children and adolescents with disabilities. Overall, the study is interesting, however there are some clarifications needed.

Comment#1
The authors should provide information about sample size estimation in this study.

**Response from Alencar et al.**

- Thank you for pointing out this need.

- Considering that it is a qualitative research, the sample was not estimated as a representation of the population, but rather an intentional selection of individuals capable of providing relevant information.
- Please see **Materials and method section/Participants and Eligibility Criteria [Ln. 162-166, P.6]**.

*The mentioned rehabilitation centers accommodated approximately 90 individuals with the potential to participate in the study. All of them were contacted; however, 27 participants met the inclusion criteria and responded to the semi-structured questionnaires and interviews I and II, after signing consent forms and authorization for images and audios.*

Comment#2
The authors should provide appropriate information about the psychometrics properties of the “My Abilities First Tool”.

**Response from Alencar et al.**

- Thank you for your suggestion.The emergence of the tool is recent, and specific psychometric studies on the MAF have not yet been conducted. As a result, there are limitations, and caution is needed when interpreting the results obtained, since its reliability and validity have not been established.
- We ensure the inclusion of appropriate information about the "MAF Tool" in the revised manuscript.
- We would like to mention that there is also a text about the tool in the Materials and Methods/My Abilities First Tool section **[Ln. 220 - 239, P. 9]**.
- In response to this comment, we inserted a concise description of the MAF tool in the Introduction section **[Ln. 90-100, P. 3 and 4]**. We added the reference Liao et al., 2023. Please see below.

*In this regard, the educational tool My Abilities First (MAF), conceived by Verònica Schiariti and disseminated in 2020, aims to achieve these measures of attention to people with disabilities. MAF is grounded in the use of positive language and the process of informing professionals, family members, and the general public about the importance of positive attitudes towards people with disabilities [6]. According to Schiariti (2020a), the use of positive language, associated with the MAF approach, means paying attention to what the person with a disability can do. MAF proposes the adoption of a skills identification card, the My Abilities ID CARD [2,6]. Furthermore, it is a proposal that, by valuing the skills of children and adolescents with disabilities, can contribute to increased community engagement and effective participation [6,8,9,10].*

Comment#3
The authors should state limitations of this study.

**Response from Alencar et al.**

- Thank you for bringing this to our attention. The information has been corrected in the revised version.
- Please in the **Final Considerations [Ln. 663 - 670, P. 28 and 29]**.

*Based on these results and considering the limitations outlined in the study, the lack of greater diversity in the sample in terms of cultural, socioeconomic, and primarily geographical differences, as well as the absence of cross-cultural adaptation of the MAF tool, may have affected obtaining a better perspective on the results. These two aspects may be crucial for promising directions in future research.*

*Furthermore, other important perspectives to be included in future studies include the formulation of strategies for the use of the My Abilities ID CARD and the development of protocols or implementation manuals for the MAF.*

6. PLOS authors have the option to publish the peer review history of their article ([what does this mean?](https://journals.plos.org/plosone/s/editorial-and-peer-review-process#loc-peer-review-history)). If published, this will include your full peer review and any attached files.

If you choose “no”, your identity will remain anonymous but your review may still be made public.

Do you want your identity to be public for this peer review? For information about this choice, including consent withdrawal, please see our [Privacy Policy](https://www.plos.org/privacy-policy).

Reviewer #1: No

Reviewer #2: No

Reviewer #3: No
